# Regulation of the Epithelial Adhesion Molecule CEACAM1 Is Important for Palate Formation

**DOI:** 10.1371/journal.pone.0061653

**Published:** 2013-04-17

**Authors:** Junko Mima, Aya Koshino, Kyoko Oka, Hitoshi Uchida, Yohki Hieda, Kanji Nohara, Mikihiko Kogo, Yang Chai, Takayoshi Sakai

**Affiliations:** 1 Department of Oral-facial Disorders, Osaka University Graduate School of Dentistry, Suita, Osaka, Japan; 2 First Department of Oral and Maxillofacial Surgery, Osaka University Graduate School of Dentistry, Suita, Osaka, Japan; 3 Section of Pediatric Dentistry, Department of Oral Growth and Development, Fukuoka Dental College, Fukuoka, Japan; 4 Department of Biology, Osaka Dental University, Kuzuha, Osaka, Japan; 5 Center for Craniofacial Molecular Biology, Ostow School of Dentistry, University of Southern California, Los Angeles, California, United State of America; Childrens Hospital Los Angeles, United States of America

## Abstract

Cleft palate results from a mixture of genetic and environmental factors and occurs when the bilateral palatal shelves fail to fuse. The objective of this study was to search for new genes involved in mouse palate formation. Gene expression of murine embryonic palatal tissue was analyzed at various developmental stages before, during, and after palate fusion using GeneChip® microarrays. *Ceacam1* was one of the highly up-regulated genes during palate formation, and this was confirmed by quantitative real-time PCR. Immunohistochemical staining showed that CEACAM1 was present in prefusion palatal epithelium and was degraded during fusion. To investigate the developmental role of CEACAM1, function-blocking antibody was added to embryonic mouse palate in organ culture. Palatal fusion was inhibited by this function-blocking antibody. To investigate the subsequent developmental role of CEACAM1, we characterized *Ceacam1*-deficient (*Ceacam1* ^−/−^) mice. Epithelial cells persisted abnormally at the midline of the embryonic palate even on day E16.0, and palatal fusion was delayed in *Ceacam1* ^−/−^ mice. TGFβ3 expression, apoptosis, and cell proliferation in palatal epithelium were not affected in the palate of *Ceacam*1^−/−^mice. However, CEACAM1 expression was retained in the remaining MEE of TGFβ-deficient mice. These results suggest that CEACAM1 has roles in the initiation of palatal fusion via epithelial cell adhesion.

## Introduction

Various cellular and molecular mechanisms underlie the elevation and fusion of the palatal shelves that are integral to normal mammalian palate formation [1]. The formation of the mammalian secondary palate requires several developmental steps, including growth, elevation, and midline fusion of the palatal shelves [2]. Two palatal shelves grow from the internal surfaces of the maxillary primordia once development of the secondary palate is initiated, and at embryonic day 13.5 (E13.5) in the mouse, they appear vertically on each side of the tongue. They subsequent elevate to a horizontal position above the tongue at E14.0 [2]. From E14.0 to E14.5, the palatal shelves make contact and start the fusion necessary for correct morphogenesis [3], and by E14.5 they are fused with one another from the middle region to the anterior and posterior regions to transform the medial edge epithelium (MEE) into the midline edge epithelial seam (MES) [4]. Ultimately, by E15.5, the MES is absent from the fused palate.

Previous studies have revealed that cleft of the secondary palate originates from a failure of signaling molecules and their receptors to control palatal shelf growth, elevation, and fusion involving palatal mesenchyme and epithelium [5], [6]. In the fusion process, most studies have focused on the mechanisms responsible for the disappearance of the MES; there still remains considerable disagreement regarding the fate of the MES, such as : (A) apoptosis in the MES [7], (B) migration of the MEE resulting in loss of MES [8], and/or (C) epithelial-mesenchymal transformation of MES [9], [10], [11]. On the other hand, before the process of disappearance of the MES, epithelial adhesion of the MEE by each opposing palatal shelf is required. However, only a few studies have investigated the initial adhesion of palatal shelves [12].

Transforming growth factor β (TGFβ) signaling plays an important role in both epithelium and mesenchyme during palate formation [13], [14]. Both TGFβ1 and TGFβ3 are normally expressed in the MEE cells of the palatal shelf during mouse palate development, whereas TGFβ2 is expressed in the mesenchyme beneath the MEE cells [15]. In *Tgfb2*-deficient (*Tgfb2^−/−^*) mice, some of the newborns (23%) exhibit cleft palate [16]. *Tgfb3^−/−^* mice show complete phenotype penetrance of cleft palate (100%) [17]. The failure of palatal shelf fusion in *Tgfb3^−/−^*mice can be rescued by exogenous TGFβ3 in an *in vitro* organ culture system [14]. Subsequent studies indicate that TGFβ3 is required for the disappearance of the MEE by inducing programmed cell death [18]. Concerning its receptor, epithelial-specific conditional knockout of *Tgfbr2* also resulted in partial cleft palate [19]. These two mouse models are useful to examine the mechanism of palatal fusion.

In the present study, we performed microarray analysis of palatal processes to identify new candidate genes that are important for palate formation. Our results provide evidence that carcinoembryonic antigen-related cell adhesion molecule 1 (CEACAM1) is expressed in a temporal manner in the palatal epithelia during palatal formation. CEACAM1 is displayed on epithelial cells of reproductive tissues such as the uterus, the breast, and the prostate [20]. In these tissues, CEACAM1 acts as a cell adhesion molecule, an angiogenic factor, a tumor suppressor, and a signal regulatory protein [21]. However, it has never been shown that CEACAM1 plays a role in craniofacial development, especially in palatogenesis. In this study, we demonstrate the role of CEACAM1 in the palatal adhesion process during palatal development.

## Materials and Methods

### Animals

All experimental procedures were approved by the Animal Use and Care Committee of the Osaka University Graduate School of Dentistry. Mature female ICR mice were obtained from Japan SLC Inc. *Ceacam1^−/−^* mice were generated as described previously [22] and were bred on the BALB/c background; these mice were kindly provided to us and maintained in the laboratory of Nicole Beauchemin. *K14-Cre*;*Tgfbr2^fl/fl^*, and *Tgfb3^−/−^* mice were generated as previously described [14], [19]. Embryonic day 0 (E0) was the day when the vaginal plug was found.

### Organ culture

The maxillary portion was removed from each fetus at E14 as previously described [23]. The explants cultured in a bottle containing BGJb medium (Invitrogen) with 1% penicillin/streptomycin (Invitrogen) (1 ml/explant) and flushed for 2 min with a gas mixture of 95% O_2_/5% CO_2_, sealed, and cultured in suspension [23], [24]. The bottles were incubated at 37°C on a roller device at 25–30 rpm for up to 60 h. For microarray analysis, the edge of the palatal processes was microdissected and collected using a microscope and forceps before, during and after palatal fusion (5 samples each). To examine the role of CEACAM1 in palatal fusion, dissected maxillary tissues were cultured in a bottle containing BGJb medium with non-specific IgG1 mAb or mouse monoclonal antibody against CEACAM1, CC1 mAb (100 µg/ml kindly provided by Dr. Kathryn V. Holmes, University of Colorado School of Medicine) [25]. The cultures were maintained for up to 60 h. Success of palatal fusion of each explant was determined by visual inspection with gentle pulling with forceps under a dissecting microscope. Fused samples were then fixed and processed for histological analysis. If any MES was present, we judged the sample as “not fused”. At least 10 serial specimens were examined by a “blinded” observer for each data point.

### RNA preparation

Total RNA was extracted from the edge of the palatal shelves before fusion and from the median tissue of the palate during and after fusion. RNA was subjected to microarray analysis and quantitative real-time RT-PCR. Palates before, during, and after fusion were prepared individually, and total RNA was extracted from each using Trizol (Invitrogen). An Agilent 2100 Bioanalyzer (Agilent Technologies) confirmed the quality and quantity of total RNA.

### Affymetrix GeneChip® microarray analysis

Changes in genome-wide gene expression profiles during palatal fusion were detected using the Mouse Genome 430 2.0 GeneChip array (Affymetrix). One µg of total RNA from each tissue sample was reverse transcribed using a One-Cycle cDNA Synthesis Kit (Affymetrix). Biotin-labeled cRNA was synthesized using a GeneChip IVT Labeling Kit (Affymetrix). Biotin-labeled cRNA was hybridized according to the GeneChip Expression Analysis Technical Manual, Rev.5 (Affymetrix). Each GeneChip was scanned using GeneChip Scanner 3000 7 G. The data were analyzed using GeneChip Operating Software (Affymetrix). Background was corrected using the Microarray Suite (MAS5.0) algorithm, and the signal value was calculated. GeneSpring GX 7.3.1 (Agilent Technologies) was used for comparative analyses. Microarray data were deposited to NCBI, Gene Expression Omnibus (Accession number: GSE43651).

### Quantitative real-time RT-PCR

Some genes were highly expressed during and after fusion. Gene expression was confirmed by quantitative real-time PCR (qPCR). Total RNA was extracted and reverse-transcribed using Superscript II. First-strand cDNA was used for qPCR using the MyiQ real-time detection system (BioRad). Melt-curve analysis and control cDNA confirmed that single products were amplified with similar efficiencies. Gene expression was normalized to *Gapdh*, reactions were performed in triplicate, and experiments were repeated at least 3 times. The qPCR was performed using the following primers.

*Ceacam1*:

5′-AGTTCCAGCATGGAGCCTGTG-3′

5′-TCCTGAGAGTGCAGGGCAGA-3′

*Gapdh*:

5′-TGTGTCCGTCGTGGATCTGA-3′

5′-TTGCTGTTGAAGTCGCAGGAG-3′

### Histological analysis and immunohistochemical microscopy

Embryonic mouse heads were fixed in 4% paraformaldehyde-PBS at 4°C. To confirm palatal fusion at the histological level, the heads were embedded in paraffin. Coronal sections (7 µm thickness) were prepared serially from anterior to posterior, and mounted on poly-L-lysine-coated slides. Sections were processed for hematoxylin and eosin staining. Palatal fusion was confirmed if there was an absence of epithelium retained in the midline of the palate. For immunostaining, sections (4 µm thickness) were treated with 4% hydrogen peroxide and incubated with mAb CC1 (1∶2000) [25], which recognizes an extracellular domain of CEACAM1. The secondary antibodies were biotinylated rabbit anti-mouse IgG polyclonal antibody (DAKO) and goat anti-rabbit IgG (Alexa Fluor 488; Invitrogen, Life Technologies). Immunoreactivity was visualized using the avidin-biotin-complex method and diaminobenzidine (DAKO) for transmitted light microscopy. Rabbit polyclonal antibody against TGFβ3 (Santa Cruz) or rabbit polyclonal anti-cytokeratin broad-spectrum screening antibody (Dako Cytomation) were used as the primary antibody, and goat anti-rabbit IgG (Alexa Fluor 488; Invitrogen Life Technologies), was used as the secondary antibody. Nuclei were counterstained by DAPI (Vectashield).

### Cell proliferation and cell death assays

Cell proliferation was determined using an anti-Ki67 antibody-proliferation marker (Abcam), and cell death was determined by terminal deoxynucleotidyl transferase-mediated deoxyuridine triphosphate nick end-labeling (TUNEL) analysis using *in situ* Apoptosis Detection Kit (TaKaRa), according to the manufacturer's instructions. The sections were then viewed under 40X magnification, and the number of proliferative cells per palate was counted.

### Statistical analysis

Results are reported as mean ± s.e.m. The comparison of different groups was performed by two-tailed Student's t-test or one-way ANOVA with the Tukey post test for experiments with more than two groups. Differences were considered statistically significant at P<0.05.

## Results

### Global Gene Expression Profiling During Palatal Fusion

**Figure 1:**
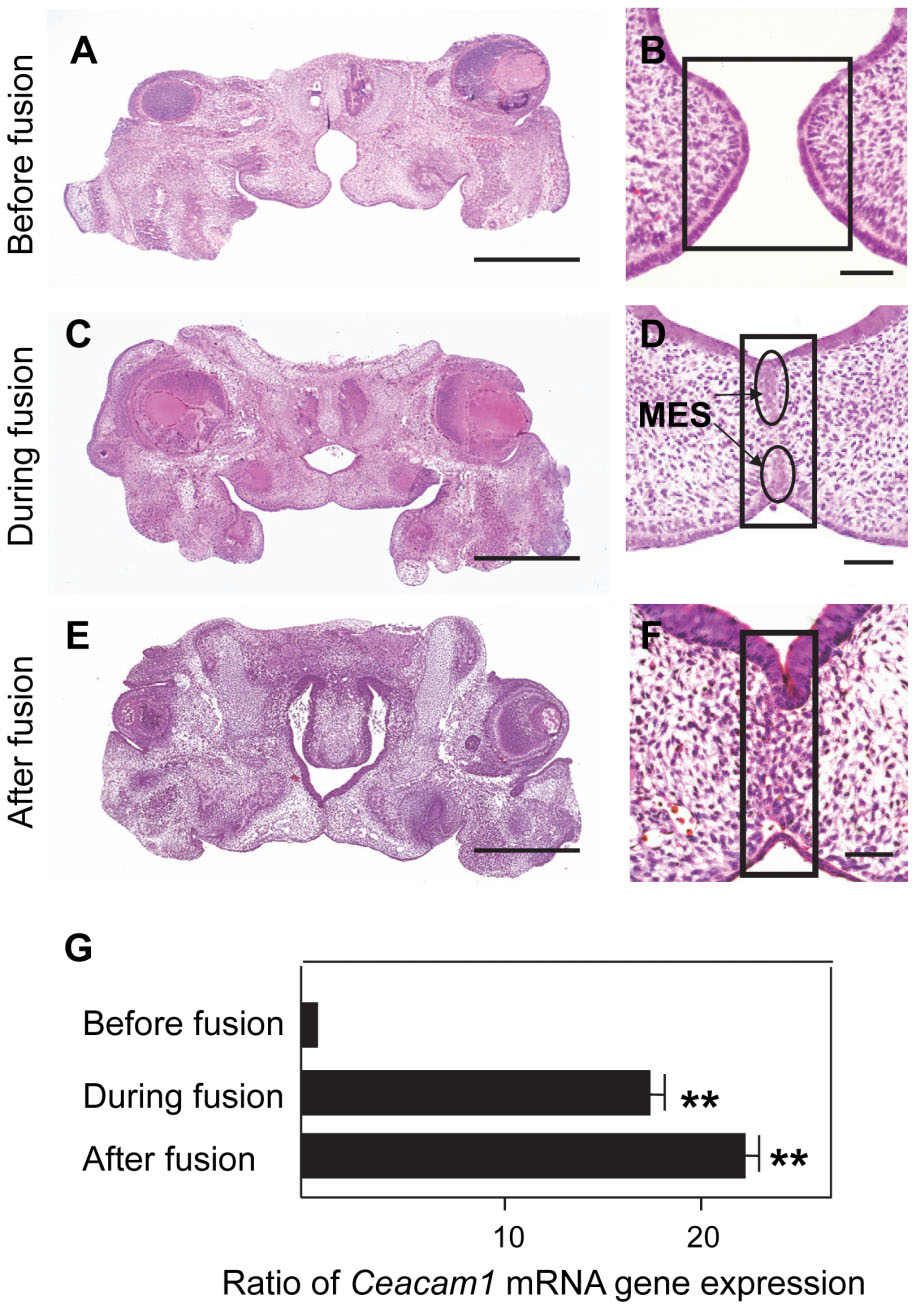
Mouse palatal fusion during palatal development in organ culture and microdissection for microarray analysis. Opposing palatal shelves had no contact in the midline after 5 h in suspension organ culture (A, B). The medial epithelial seam (MES) was detected in fusing palatal shelves after culture for 24 h (C, D). The MES was disappeared completely after 60 h cultured (E, F). Tissues were excised from the boxed regions at each stage for microarray analysis and qPCR (B, D, F). Scale bar, 500 µm (A, C, E), 50 µm (B, D, F). The qPCR analysis confirmed the expression of *Ceacam1* mRNA in the boxed regions before, during, and after fusion (G). *Ceacam1* mRNA expression levels during fusion (17.2±2.1) and after fusion (22.2±2.0) were normalized to before fusion. Bars indicate s.e.m. **P<0.01 compared with control (before fusion).

In palate formation, the palatal shelves originally lay vertically at E13.5. At E14.0, the shelves elevated horizontally and became juxtaposed before palate fusion [3], [12]. By E14.5, the medial edge epithelium (MEE) formed the midline edge epithelial seam (MES) during fusion [4]. At E15.0, the MES was disrupted, and epithelial islands were observed, which a complete disappearance at E15.5 after fusion. Here we showed that histological changes of palatal formation with suspension cultured were shown in Figure 1A–F. The prefused each palatal shelves became to be close easily due to absence of tongue (Figure 1A, B). After attached the each palatal shelves, fusing process started. The MES was detected during fusion as well as normal pathogenesis at E14.5 (Figure 1C, D). It was taken for 60 h to completely disappear of MEE in the midline of palate (Figure 1E, F). Our suspension culture methods were clearly demonstrated the process of palatogenesis in *ex vivo* situation. In order to identify candidate molecules of palatal fusion, palatal tissues were dissected for microarray analysis as shown in the boxes areas of Figure 1B, D, F. Microarray analysis was performed to detect the gene expression of palatal tissues at various stages, including before, during, and after palatal fusion. The number of genes up-regulated and down-regulated more than 2-fold were 3663 and 2947, respectively. Large changes were seen in the expression of *Krt13* (keratin 13), *Ceacam1*, *Car3* (carbonic anhydrase 3), *Fmo2* (flavin containing monooxygenase 2), *Hdc* (histidine decarboxylase), and *Armc3* (armadillo repeat containing 3) (Table 1). One of the highly up-regulated genes was *Ceacam1*. CEACAM1 is known as an adhesion and angiogenic molecule. We chose to study this molecule further because epithelial cell adhesion is an extremely important process for the initiation of palatal fusion.

**Table 1:** Highly expressed genes in microarray analysis of embryonic palatal tissue during and after palatal fusion.

Gene name	Ratio of Gene Expression (normalized to before fusion)		
	Before fusion	During fusion	After fusion
*Krt13*	1.0	112.6	290.7
*Ceacam1*	1.0	38.0	106.1
*Car3*	1.0	37.4	17.2
*Fmo2*	1.0	32.1	65.4
*Hdc*	1.0	26.4	673.2
*Armc3*	1.0	21.5	276.9

### *Ceacam1* Expression Confirmed by Quantitative Real-time PCR

*Ceacam1* was expressed at a very low level in the palate before fusion, but was higher expressed in the midline of the palate during and after fusion based on the microarray data. To confirm this result, qPCR was also performed using palatal tissue before, during, and after fusion after microdissection as shown in Figure 1 A–F. The qPCR demonstrated that *Ceacam1* had important role during palate formation. These data supported the result of microarray analysis (Figure 1G).

### Expression of CEACAM1 in Developing Embryonic Craniofacial Tissue

**Figure 2:**
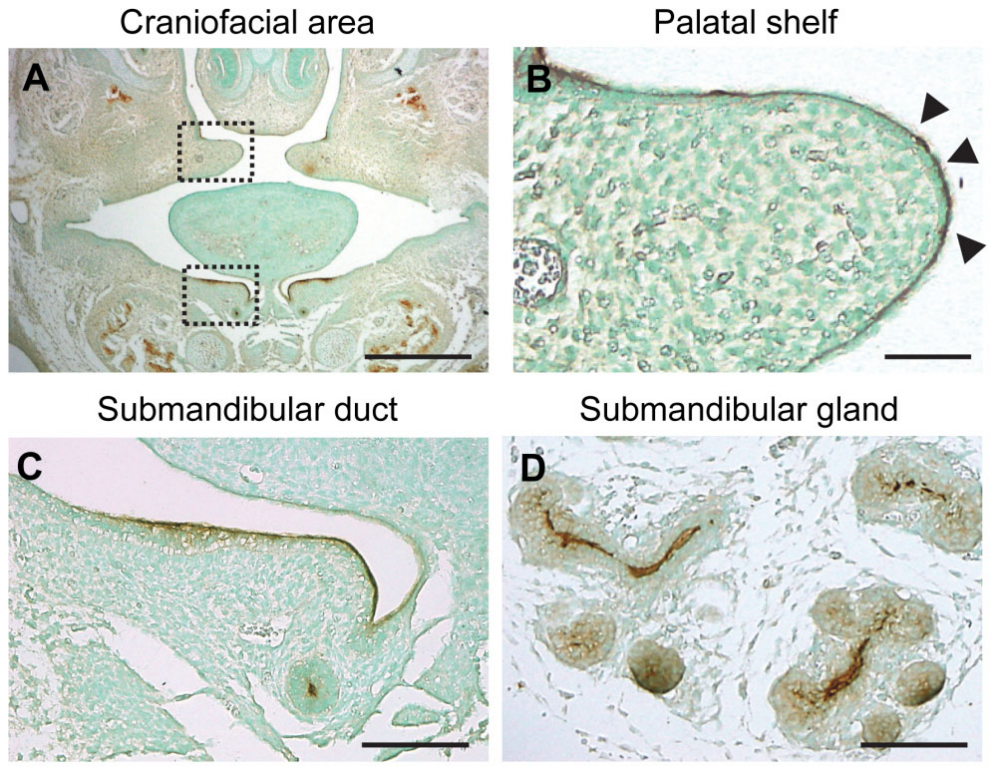
Expression of CEACAM1 in embryonic developing craniofacial tissue. CEACAM1 expression in the craniofacial region in wild-type embryo at E14.0 (A). Dotted rectangles show the medial edges of the epithelium and submandibular duct. These areas are shown enlarged (B, C). CEACAM1 was expressed in the epithelia of pre-fusion palatal shelf (B, arrowhead), submandibular duct (C) and submandibular gland (D). Scale bar, 200 µm (A), 50 µm (B), 100 µm (C, D).

To observe the distribution of CEACAM1 in embryonic craniofacial tissue, immunohistochemical analysis was performed. At E14.5, weak expression of CEACAM1 was observed in the epithelium of developing craniofacial tissue (Figure 2A–D). CEACAM1 was expressed in the apical side of medial edges of the epithelium (Figure 2B). In submandibular duct and glands, it was similarly expressed in the apical epithelium (Figure 2C, D). These results demonstrate that CEACAM1 showed characteristic expression in craniofacial tissues such as palate and salivary gland epithelium.

### Distribution and Role of CEACAM1 in Palatal Tissue

**Figure 3:**
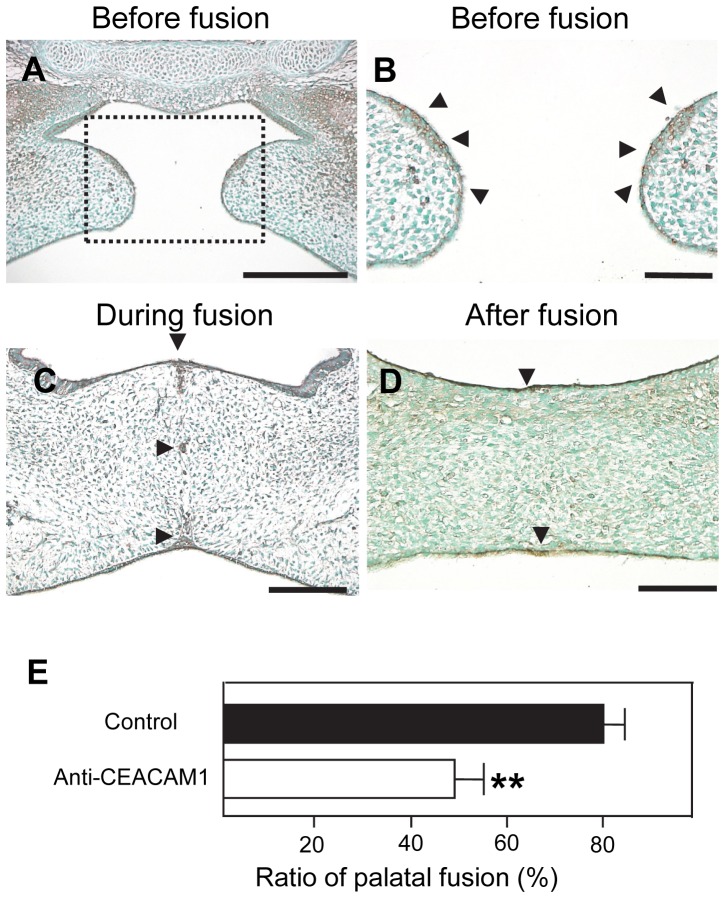
Expression of CEACAM1 and effect of anti-CEACAM1 antibody (mAb CC1) on palate fusion. CEACAM1 expression in palatal shelves before fusion (A). Enlarged image of dotted rectangle area shown in A (B). CEACAM1 was expressed in the MEE before fusion (B), and was retained in the MES during fusion (C). The MES was not observed after fusion. CEACAM1 was not seen in the center of palatal shelves, but was expressed in the nasal and oral epithelium (D). Arrowheads indicate the distribution of CEACAM1 expression in palatal epithelium (B, C and D). Scale bar, 100 µm (A), 50 µm (B, C, D). To examine the role of CEACAM1, palatal shelves were cultured for 60 h with control (non-specific IgG) and mAb CC1 (anti-CEACAM1 antibody). The frequency of palatal fusion was calculated as the percentage of fused palatal shelves relative to the total number analyzed. The mAb CC1 inhibited palatal fusion (E); the frequency of palatal fusion in the presence of mAb CC1 was 48.3%±6.7%, while control fusion frequency was 79.8%±5.6%. Bars indicate s.e.m. **P<0.01 compared with control.

Immunohistochemical analysis showed that CEACAM1 was expressed in the palatal epithelium. Therefore, we performed immunohistochemical analysis of CEACAM1 expression before, during and after fusion using wild-type mice. Before the fusion of palatal shelves, CEACAM1 was expressed in the MEE (Figure 3A, B). During fusion, the MES retained some CEACAM1 expression (Figure 3C). After fusion, there were no epithelial components nor any CEACAM1 expression observed in the center of the palatal shelves. Thereafter, CEACAM1 was only expressed in the nasal and oral epithelium in the midline (Figure 3D). We further investigated the functional significance of CEACAM1 expression during palate formation using a suspension culture system. In this organ culture system, the function-blocking antibody mAb CC1 [25], [26] was added to the medium, and the palatal tissues were cultured for 60 h. Initial palatal adhesion and fusion were inhibited by antibody blocking of CEACAM1. Control (non-specific IgG) showed a normal high score of palatal fusion (79.8±5.6%), whereas palatal fusion was significantly inhibited by mAb CC1 (48.3±6.7%) (Figure 3E). This result demonstrates the direct influence of CEACAM1 on the initial adhesion process during palatal fusion.

### Palate Fusion in the *Ceacam1* ^−/−^ Embryonic Mouse

**Figure 4:**
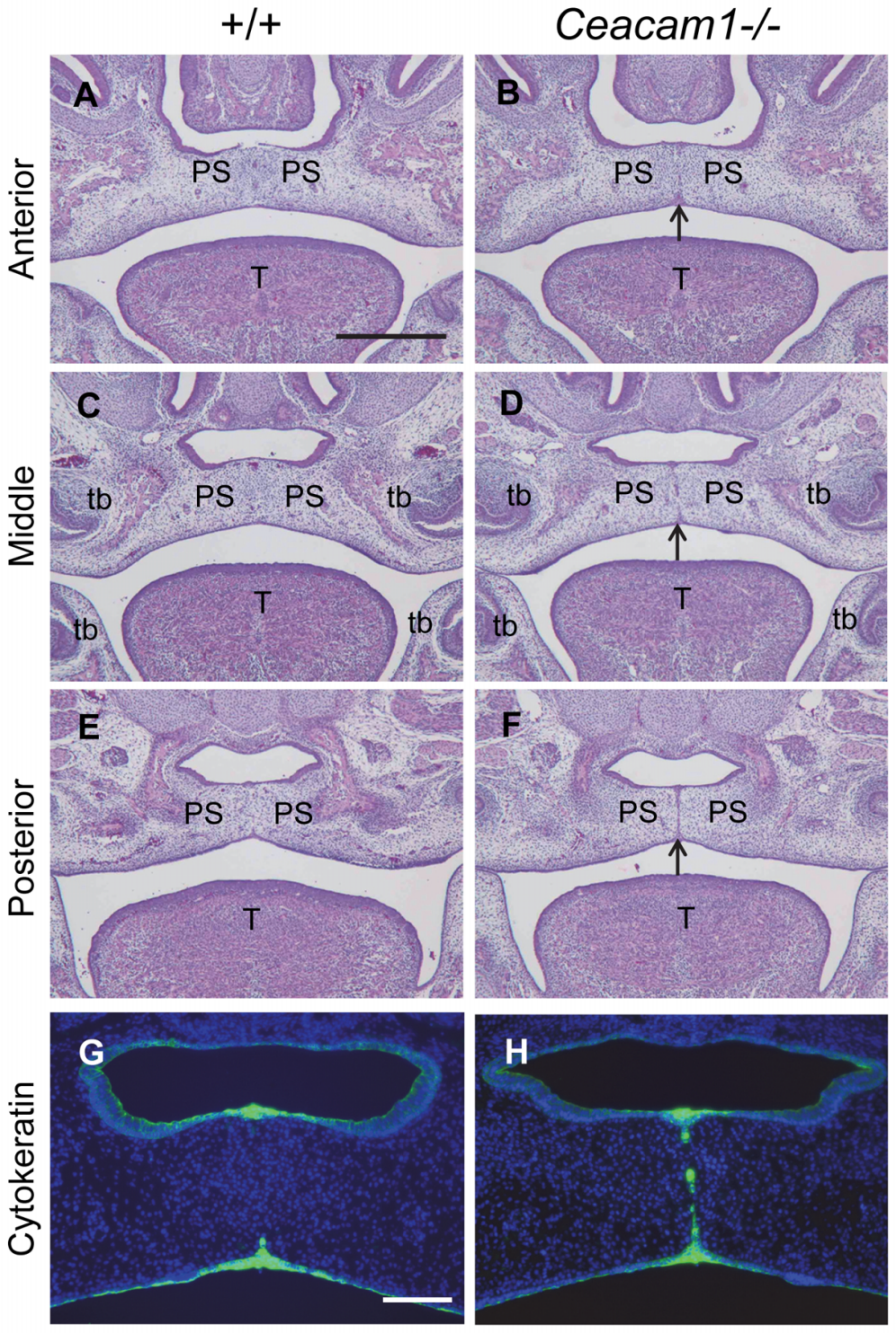
*Ceacam1* deficiency causes partial cleft secondary palate. At E15.5, palatal fusion was well under way in the anterior, middle and posterior regions of palatal shelves in the wild-type embryo. There was no epithelial component at the midline of the palate (A, C, E). The *Ceacam1* mutant embryo shows the MES in the area of palatal fusion (arrow) (B, D, F). Cytokeratin staining supported the existence of the MES, indicating a delay of palatal fusion (G, H). PS, palatal Shelf; T, tongue; tb, tooth bud. Scale bar, 200 µm (A–F), 50 µm (G, H).

Based on the previous results, we hypothesized that the adhesion molecule *Ceacam1* has an important role of the epithelial fusion of palatal shelves. Consequently, we evaluated the histological appearance of palatogenesis in *Ceacam1* ^−/−^ mice overall, there were few epithelial cells remaining at the fusing midline of palates of wild-type mice at E15.5 (Figure 4A, C, E, G). In contrast, in *Ceacam1* ^−/−^ palates at this same stage, the MES still remained (Figure 4B, D, F, H). Cytokeratin staining is helpful to visualize the MES using the same sliced section (Figure 4G, H). Contrary to our expectations, palatal fusion did eventually occur, even though delayed in the *Ceacam1* ^−/−^ mice. This finding suggested that *Ceacam1* expression in MEE may relate to the disappearance of MEE cells.

### Cell Proliferation, Cell Death, and TGFβ3 Signaling in *Ceacam1* ^−/−^Palate Fusion

**Figure 5:**
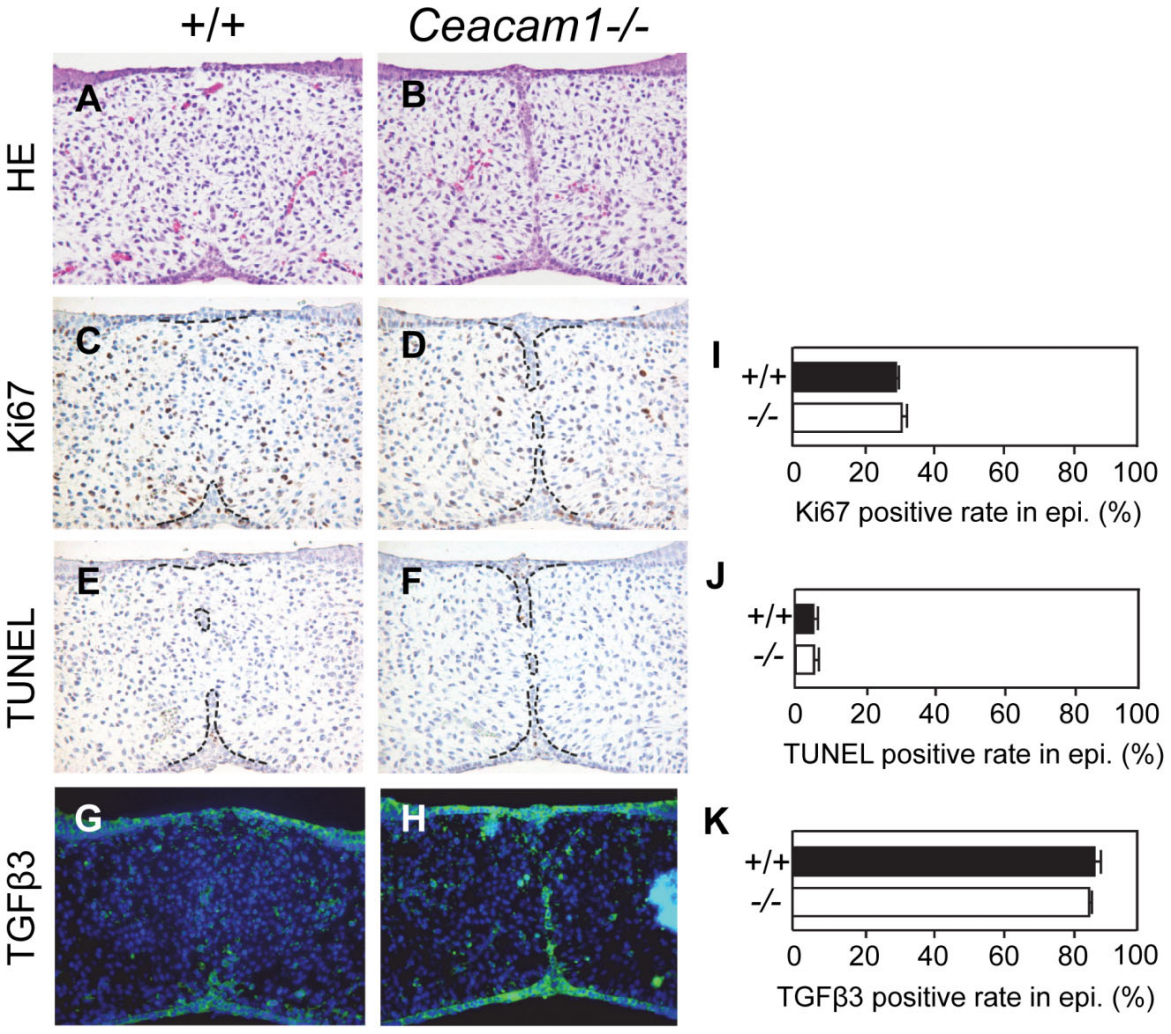
TGFβ signaling, proliferation, and apoptosis during palate formation in *Ceacam1* ^−/−^ mice. At E15.5, palatal fusion was well under way in the wild-type embryo (+/+) (A, C, E, G). *Ceacam1* ^−/−^ embryos (−/−) showed residual epithelial islands (B, D, F, H), similar to Figure 4. *Ceacam1* ^−/−^ MEE cells show positive Ki67 and TUNEL staining, markers for cell proliferation and apoptosis, respectively, from the anterior to the posterior part of the palate, and they express TGFβ3 at levels similar to the wild-type MEE cells. Expression levels were statistically analyzed (I, J, K); Ki67, 28.8±0.3 (+/+) (%), 30.6±2.4 (−/−) (%) (I); TUNEL, 5.0±1.4 (+/+) (%), 4.9±1.3 (−/−) (%) (J); TGFβ3, 86.9±3.7 (+/+) (%), 84.6±0.6 (−/−) (%) (K). Bars indicate s.e.m.; P = 0.52 (I), 0.50 (J), 0.50 (K), in *Ceacam1* ^−/−^ palates compared to wild-type (+/+).

Since the MES was retained longer in the *Ceacam1* ^−/−^ mice, we determined whether the loss of *Ceacam1* expression in the palate resulted in intrinsic changes in the disappearance of the MES. We compared the levels of cell proliferation and apoptosis at E15.5 in palates from wild-type and *Ceacam1* ^−/−^ mice (Figure 5A–H). We determined the proliferative capacities of the palatal regions in wild-type and *Ceacam1* ^−/−^ mice by Ki-67 immunohistochemical analysis. *Ceacam1* ^−/−^ mice had the same levels of Ki-67-positive palatal mesenchymal cells as wild-type mice (Figure 5C, D, I). TUNEL-positive cells were detected in *Ceacam1* ^−/−^ mice, but their numbers were at the same level as in wild-type mice (Figure 5E, F, J). Therefore, there were no significant differences in proliferation or apoptosis of either epithelial or mesenchymal compartments between the wild-type and *Ceacam1* ^−/−^ palatal shelves. *Ceacam1* ^−/−^ mice also had nearly the same levels of TGFβ3-positive cells in the MES as the controls (Figure 5G, H, K). These results suggest that CEACAM1 does not affect proliferation, apoptosis, or TGFβ3 signaling related to the disappearance of MES.

### Distribution of CEACAM1 in *Tgfb3^−/−^* and *K14Cre;Tgfbr2^fl/fl^* Mice

**Figure 6:**
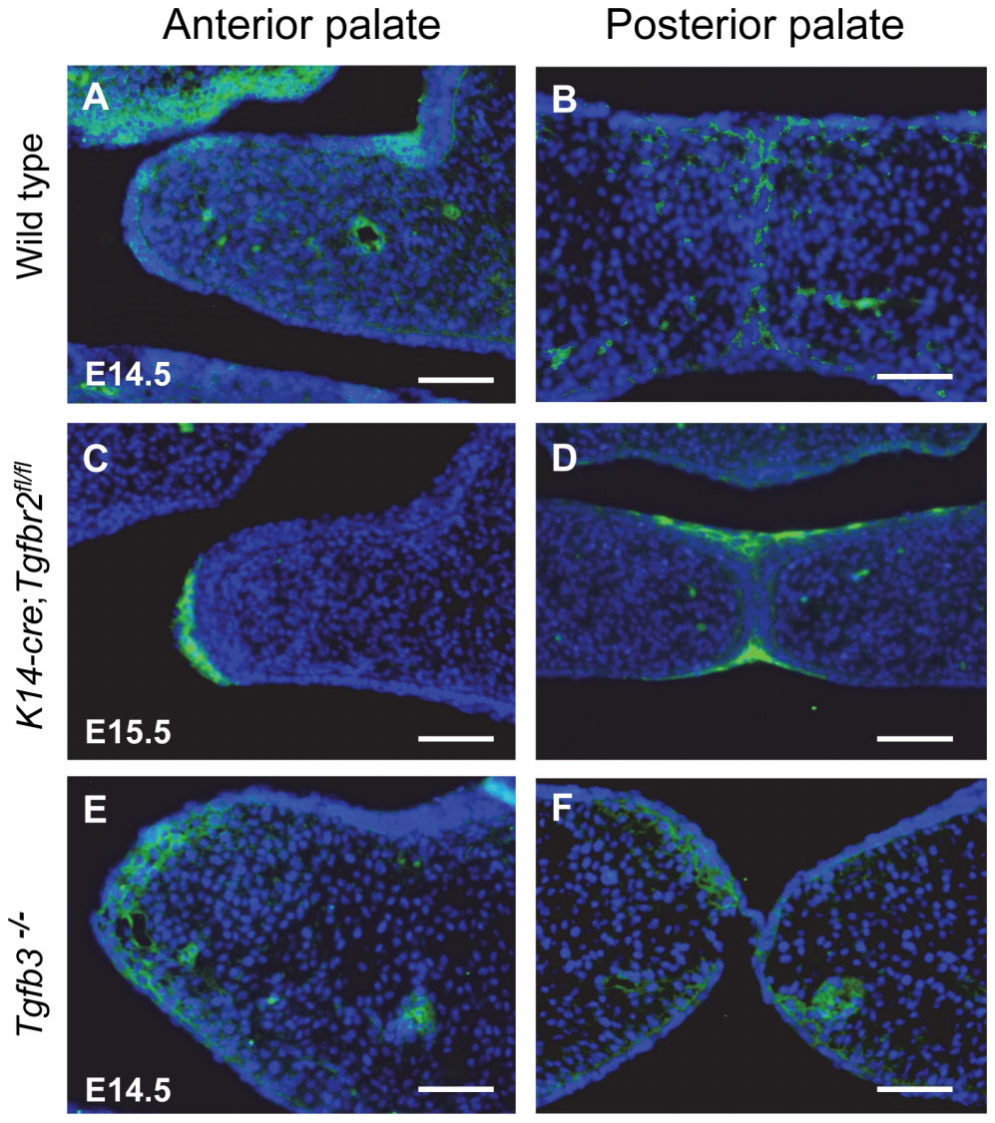
Expression of CEACAM1 in *K14-cre*;*Tgfbr2^fl/fl^* and *Tgfb3^−/−^* palatal shelves. CEACAM1 was expressed in the MEE of anterior and posterior regions of palatal shelves in wild-type embryos at E14.5 (A, B). CEACAM1 was clearly expressed in the MEE of the anterior region of the unfused palatal shelf in *K14-cre*;*Tgfbr*2*^fl/fl^* (C) and was retained in the oral and nasal triangle of MEE of at E15.5 (D). CEACAM1 was expressed in the epithelium and mesenchyme of both anterior and posterior regions of palatal shelves in *Tgfb3* ^−/−^ mice at 14.5 (E and F). Scale bar, 50 µm (A–F).

We next examined different genetically-modified mice known to have palatal epithelium defects to further define the role and expression of CEACAM1 in palate formation. Although TGFβIIR is strongly expressed in both MEE and cranial neural crest (CNC)-derived palatal mesenchyme, mice deficient for the *Tgfbr2* gene die on E10.5 as the result of defects in yolk sac hematopoiesis and vasculogenesis [27]. Conditional inactivation of *Tgfbr2* in palatal epithelium using *K14-Cre*;*Tgfbr2^fl/fl^* mice revealed incomplete disappearance of the MEE and submucous cleft in palate formation [19]. *Tgfb3^−/−^* mice also show the failure of palatal shelf fusion with 100 percent penetrance [17]. Subsequent studies indicated that TGFβ3 is required for the disappearance of MEE by inducing programmed cell death [18]. Additionally, it has been reported that TGFβ3 in palatal epithelium regulates cell-cell adhesion molecules such as E-cadherin during fusion of palatal shelves [28], [29]. Therefore, we investigated that whether CEACAM1 expression was changed in TGFβ-deficient mice, which have defects in the disappearance of MEE. We compared wild-type mice at E14.5 to *K14-Cre*;*Tgfbr2^fl/fl^* mice at E15.5 and *Tgfb3* ^−/−^ mice at E14.5. CEACAM1 expression was weakly observed in the MEE of anterior and posterior regions of palatal shelves in wild-type embryos (Figure 6A, B). Residual MES palatal epithelium was seen in *K14-Cre*;*Tgfbr2^fl/fl^* mutant embryos (Figure 6C, D). Interestingly, CEACAM1 was clearly expressed in the remaining palatal epithelium in *K14-Cre*;*Tgfbr2^fl/fl^* embryos. In *Tgfb3* ^−/−^mice, the opposing palatal shelves were just attached at the midline of the developing palate (Figure 6F). CEACAM1 expression was also retained in the palatal epithelium and mesenchyme of palatal shelves (Figure 6E, F). To investigate the effect of TGFβ3 on CEACAM1 expression, exogenous TGFβ3 beads were placed in the organ culture undergoing palatal fusion. TGFβ3 did not directly affect the expression of CEACAM1 (data not shown).

## Discussion

Recently, many genes have been studied for their roles in palate formation. Fusion of the palate occurs in three distinct steps: 1. the MEE of each palatal shelf adhere; 2. the MEE of each palatal shelf forms the MES; 3. the MES disappears. Most recent studies have focused on the mechanism of the disappearance of the MES, which is known to involve apoptosis, epithelial-mesenchymal transition (EMT), and/or cell migration. Little is known about the adhesion molecules that are critical players for attachment of opposing palatal shelves and MEE adhesion.

Cell adhesion to either a substrate or another cell is essential in development and in the maintenance of cell and tissue structure [30]. To elucidate the mechanism of palate formation, especially epithelial adhesion in craniofacial development, we performed microarray analysis to detect genes expressed in palatal tissues at various developmental stages including before, during, and after palatal fusion. We identified more than three thousand genes that are up-regulated during and after palatal fusion. We hypothesized that these up-regulated genes play important developmental roles, and started searching for functional genes that have not been previously reported. During palatal fusion, *Ceacam1*, *Armc3*, *Car3*, *Fmo2*, *Hdc*, and *Ker13* are predominately up-regulated. *Ceacam1* was identified as a candidate gene that may be important for embryonic palatal fusion.

CEACAM1, a member of the CEA family belonging to the Ig superfamily, is an adhesion molecule. It has been suggested as a candidate molecule that regulates immunological homeostasis in the intestine. Previous research reported that CEACAM1 functions in cell adhesion, apoptosis, and angiogenesis. There are also a few studies on its role in morphogenesis in the mammary gland [31]. CEACAM1 also functions as an epithelial tumor suppressor and as an angiogenic growth factor [22], [32]. These studies suggest that CEACAM1 regulates epithelial adhesion and morphogenesis. CEACAM1 expression is induced by VEGF. CEACAM1 contributes to endothelial cell tube formation (angiogenesis) as an additional effect of VEGF [33]. CEACAM1 is expressed in adherence junctions, and it supports cell adhesion and polarity [34]. CEACAM1 displays both homophilic adhesion (CEACAM1-CEACAM1) and heterophilic adhesion (CEACAM1-CEA) [35]. One hypothesis is that CEACAM1 contributes to cell adhesion and morphogenesis of the palate. However, there are no reports that indicate the distribution and role of CEACAM1 in palatal fusion.

To address the functional role of CEACAM1 in palatal fusion, we examined the expression of CEACAM1 during palatal development. CEACAM1 is expressed at the apical side of palatal epithelium before fusion and is lost along with the progressive disappearance of MEE. Interestingly, *Ceacam1* mRNA expression was increased during and after palatal fusion, even though epithelial cells of the MES had disappeared. Because CEACAM1 is expressed in nasal and oral epithelium after palatal fusion, this discrepancy is likely due to *Ceacam1* mRNA contributed by the nasal and oral epithelium. We also performed functional inhibition studies using antibody and analyzed the palatal tissues of the *Ceacam1^−/−^* mouse. The anti-functional CEACAM1 antibody inhibited palatal adhesion between opposing palatal shelves. These experiments demonstrated that CEACAM1 is very important for adhesion of palatal shelves at the initiation of palatal fusion.

Previous studies indicated that palatal fusion is related to the differentiation of epithelial tissue. The epithelial tissue that was not fusing was covered with periderm, but periderm was absent in the fusing area [12]. CEACAM1 was detected in the epithelium of palatal processes before attachment of the palatal shelves. Consequently, CEACAM1 may have some function in adhesion through the formation of periderm in palatal epithelium. If *Ceacam1* is an absolutely critical factor for initial adhesion of palatal shelves, we hypothesized that the loss of *Ceacam1* in palatal epithelium would result in complete failure of palatal fusion. However, palatal fusion of *Ceacam1^−/−^*mice was only delayed compared with wild type mice, indicating a role in initial adhesion but not an absolute requirement for eventual fusion.

Apoptosis is the major process for the decrease in MEE during palatal fusion. To explore the mechanism responsible for the failure of MEE disappearance in the *Ceacam1^−/−^*mice, we investigated whether there was any difference in apoptosis. The fate of MEE showed no change in the *Ceacam1^−/−^* palate. Wild-type MEE cells showed positive TUNEL staining as a marker for cell death from the anterior to the posterior part of the palate. At E15.5, the palate fusion process was completed, and most of the wild-type MEE cells had disappeared. *Ceacam1^−/−^* MEE cells still maintained the ability to proliferate and were positive for Ki67 staining throughout the entire palate. We concluded that palatal fusion in the *Ceacam1^−/−^*mouse was delayed, but cell proliferation and apoptosis were not affected. Therefore, we suggest that the loss of initial cell adhesion in *Ceacam1^−/−^* leads to a delay of palatal fusion without changes in proliferation or apoptosis.

Analysis of the CEACAM1 levels in two different genetically modified models with various forms of cleft palate gave us a further indication of how CEACAM1 might be functioning. Ablation of *Tgfbr2* in palate epithelial cells in *K14-cre*;*Tgfbr2^fl/fl^* mice showed a soft palate cleft, submucosal cleft, and failure of the primary palate to fuse with the secondary palate [19]. *Tgfb3^−/−^* mice show complete phenotype penetrance of cleft palate [14]. And recent research showed that TGFβ3 inhibits E-cadherin gene expression in palate medial-edge epithelial cells [28]. It suggested that degradation of adhesion molecules by TGFβ is critical event for disappearance of MEE during palatal fusion process. In our present study, we showed that TGFβ3 was expressed at normal levels in the epithelial cells of *Ceacam1^−/−^* mice palate. These data indicate that *Ceacam1* is likely a downstream target gene of TGFβ3 signaling rather than *vice versa*. The continued expression of CEACAM1 after loss of TGFβ signaling in the epithelium could be a reason for the cleft palate in TGFβ-signal deficient mice.

We conclude that CEACAM1 expression can be influenced by the status of TGFβ3 signaling. Its decreased expression at sites of failed palatal fusion is consistent with the antibody inhibition and gene ablation data indicating a direct role in the initiation of palate formation via epithelial cell adhesion.

Our fetal global gene discovery study on palatal fusion using microdissection and microarray analysis is the first of its kind. To the extent that epithelial adhesion is critical to palate formation, identification of new genes and pathways involved in this process will help efforts toward the development of novel strategies to protect and enhance palate fusion and improve treatments for cleft palate.
